# Small *Klebsiella pneumoniae* Plasmids: Neglected Contributors to Antibiotic Resistance

**DOI:** 10.3389/fmicb.2019.02182

**Published:** 2019-09-20

**Authors:** Maria S. Ramirez, Andrés Iriarte, Rodrigo Reyes-Lamothe, David J. Sherratt, Marcelo E. Tolmasky

**Affiliations:** ^1^Center for Applied Biotechnology Studies, Department of Biological Sciences, College of Natural Sciences and Mathematics, California State University Fullerton, Fullerton, CA, United States; ^2^Laboratorio de Biología Computacional, Departamento de Desarrollo Biotecnológico, Facultad de Medicina, Universidad de la República de Uruguay, Montevideo, Uruguay; ^3^Department of Biology, McGill University, Montreal, QC, Canada; ^4^Department of Biochemistry, University of Oxford, Oxford, United Kingdom

**Keywords:** transposon, integron, plasmid, ESKAPE, *Klebsiella*, multidrug resistance

## Abstract

*Klebsiella pneumoniae* is the causative agent of community- and, more commonly, hospital-acquired infections. Infections caused by this bacterium have recently become more dangerous due to the acquisition of multiresistance to antibiotics and the rise of hypervirulent variants. Plasmids usually carry genes coding for resistance to antibiotics or virulence factors, and the recent sequence of complete *K. pneumoniae* genomes showed that most strains harbor many of them. Unlike large plasmids, small, usually high copy number plasmids, did not attract much attention. However, these plasmids may include genes coding for specialized functions, such as antibiotic resistance, that can be expressed at high levels due to gene dosage effect. These genes may be part of mobile elements that not only facilitate their dissemination but also participate in plasmid evolution. Furthermore, high copy number plasmids may also play a role in evolution by allowing coexistence of mutated and non-mutated versions of a gene, which helps to circumvent the constraints imposed by trade-offs after certain genes mutate. Most *K. pneumoniae* plasmids 25-kb or smaller replicate by the ColE1-type mechanism and many of them are mobilizable. The transposon Tn*1331* and derivatives were found in a high percentage of these plasmids. Another transposon that was found in representatives of this group is the *bla*_KPC_-containing Tn*4401*. Common resistance determinants found in these plasmids were *aac(6′)-Ib* and genes coding for β-lactamases including carbapenemases.

## Introduction

*Klebsiella pneumoniae*, although usually carried by healthy humans, is also the causative agent of community- and, more commonly, hospital-acquired infections, accounting for more than 30% of those caused by Gram-negative bacteria (Kalpoe et al., 2012; Shon et al., 2013; Li et al., 2014; Calfee, 2017; Navon-Venezia et al., 2017). *K. pneumoniae* became more dangerous in recent years due to the acquisition of multidrug resistance (Almaghrabi et al., 2014; Chen et al., 2014a; Ramirez et al., 2016; Rojas et al., 2017b) and the emergence of hypervirulent variants (Decre et al., 2011; Kalpoe et al., 2012; Shon et al., 2013). The most common diseases in Western countries, caused by classic (non-hypervirulent) strains, are urinary tract infections, pneumonia, septicemias, meningitis, and soft tissue infections (Shon et al., 2013; Ramirez et al., 2016; Calfee, 2017; Martel et al., 2017; Gupta et al., 2018; Osman et al., 2018). *K. pneumoniae* may also play a role in ankylosing spondylitis and Crohn’s disease (Rashid et al., 2013, 2016). Hypervirulent variants overproduce capsular polysaccharide and are hypermucoviscous, a phenotype defined when an inoculation loop or needle generates a viscous string >5 mm in length by stretching bacterial colonies on an agar plate (Shon et al., 2013). They are characterized for showing metastatic spread and causing life-threatening community-acquired infections like liver abscess, pneumonia, osteomyelitis, meningitis as well as endophthalmitis in immunocompetent healthy individuals (Cheng et al., 1991; Chen et al., 2004, 2019; Sobirk et al., 2010; Decre et al., 2011; Shon et al., 2013; Prokesch et al., 2016; Martel et al., 2017; Zhan et al., 2017; Gupta et al., 2018; Jun, 2018; Osman et al., 2018; Yao et al., 2018; Marr and Russo, 2019).

As it is the case with most bacteria, virulence factors and drug resistance traits are commonly encoded by *K. pneumoniae* plasmids (Nassif and Sansonetti, 1986; Nassif et al., 1989a, b; Wacharotayankun et al., 1993; Deleo et al., 2014; Ramirez et al., 2014a; Navon-Venezia et al., 2017; Peirano et al., 2017; Rozwandowicz et al., 2018; Turton et al., 2018). The recent completion and analysis of genomes of numerous *K. pneumoniae* strains clarified different characteristics of their chromosomes and showed that they usually harbor several plasmids with a broad range of sizes from a few to several hundred kb (Nassif et al., 1989a; Liu et al., 2012; Ramirez et al., 2012, 2014b, 2016; Deleo et al., 2014; Martin and Bachman, 2018). The larger plasmids are usually low copy number and may include genes and functions like antibiotic resistance, virulence factors, and conjugation properties. This rich combination of genes involved in virulence and resistance to treatment, together with the ability to disseminate these traits, attracted the majority of the efforts to study the properties and biology of *K. pneumoniae* plasmids (Chen et al., 2004; Soler Bistue et al., 2008; Garcia-Fernandez et al., 2012; Mathers et al., 2015; Conlan et al., 2016; Rojas et al., 2017a, b; Zhou et al., 2017; Desmet et al., 2018). However, although carrying a more modest number of genes, small high-copy number plasmids are of interest because they have a significant impact on bacterial infection harboring a variety of resistance genes that are expressed at high levels and that are usually included in mobile elements. These plasmids also play important, albeit still understudied, roles in plasmid evolution through events mediated by mobile elements as well as several recombinational mechanisms of cointegration with other plasmids that can be followed by imprecise resolution (Zakharova et al., 2002; Bui et al., 2006; Tran et al., 2012; Lin et al., 2013; Ramirez et al., 2014a; He et al., 2015; Cameranesi et al., 2018). Recent studies also showed that multicopy plasmids promote evolution by permitting the coexistence of novel and ancestral traits when a new variant arises through mutation, allowing bacteria to escape the evolutionary constraints imposed by the trade-offs that otherwise would limit the perpetuation of certain gene changes (Rodriguez-Beltran et al., 2018). Furthermore, the usual higher copy number of small plasmids may facilitate the enhancement of resistance levels by gene dosage (Tolmasky et al., 1988; Sandegren and Andersson, 2009).

## The pJHCMW1 Plasmid

**FIGURE 1:**
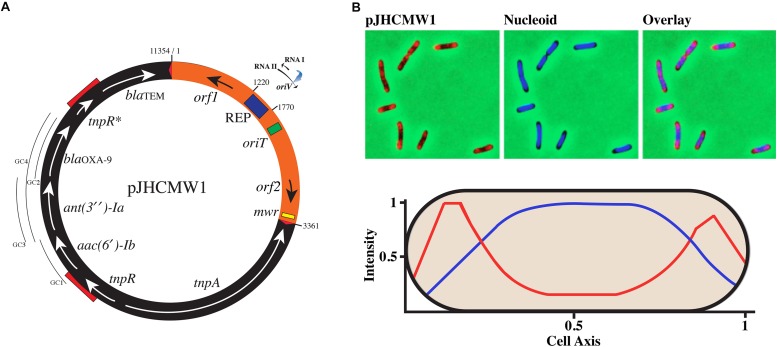
Genetic map and subcellular localization of pJHCMW1. **(A)** The numbers indicate the coordinates as defined in GenBank (accession number AF479774). The region harboring the inheritance functions is shown in orange and boxes inside indicate the location of the replication region (REP) and *oriT* and *mwr* loci. The RNA I and RNA II within the replication region are shown with their transcription orientation (arrowheads). The blue arrow represents the location of RNase H digestion where DNA polymerase I initiates replication (*oriV*). The black arrow adjacent to REP is a gene that codes for a PH domain-containing protein of unknown function. The black arrow adjacent to the *mwr* site represents a gene coding for a hypothetical protein of unknown function. The black region represents Tn*1331*. The red triangles represent the inverted repeats (GGGGTCTGACGCTCAGTGGAACGAAAACTCACGTTAAG) and the red segments on top of the circle indicate repeated sequences most probably formed during the genesis of the transposon. The segment including one of these repeated sequences plus the region between them is the addition to Tn*3* that formed Tn*1331* (Tolmasky, 2000). The lines labeled as GC1, GC2, GC3, and GC4 indicate the location of the gene cassettes. GC1 and GC2 are non-functional, GC3 is poorly functional, and GC4, which includes both *ant(3^″^)-Ia*, and *bla*_OXA–__9_, is fully functional (Ramirez et al., 2008). **(B)** The top panels show images of cells carrying the pJHCMW1 derivative pTT4 that possesses the TetO. The panel to the left shows the cells labeled using TetR-YPet to detect plasmid molecules, the center panel shows labeling with DAPI to detect the nucleoid, and the panel to the right shows an overlay of nucleoid and plasmid signals. The bottom panel shows the levels of fluorescent signals using the same colors as the top panels over a cell’s length. The values are averages of measurements from 329 cells.

**FIGURE 2:**
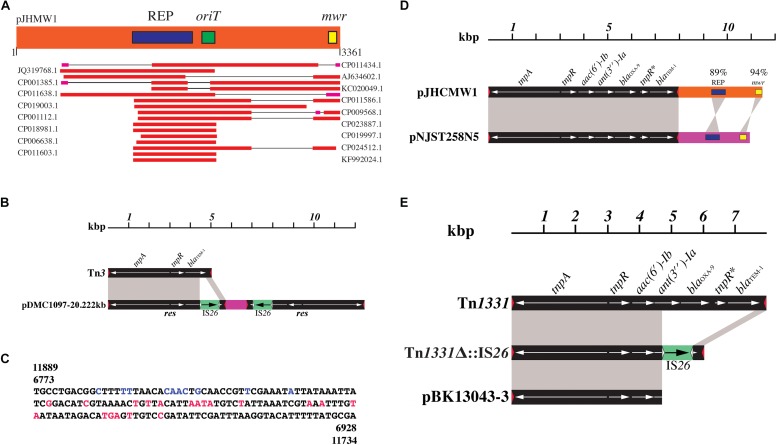
Small *K. pneumoniae* plasmids and mobile elements. **(A)** The pJHCMW1 region containing the inheritance functions (3361 nucleotides) was analyzed using BLAST and the plasmids showing homology in segments larger than the REP region are shown in the figure. Homology analysis was done using BLAST (Ncbi_Resource_Coordinators, 2018). **(B)** Comparison of the genetic maps of Tn*3* and the pDMC1097-20.222kb transposon-like structure. The nucleotide sequence of Tn*3* (accession number V00613) was compared to that of pDMC1097-20.222kb (accession number NZ_CP011979.1). Two inverted repeated structures with homology to a region at the right end of Tn*3* are present in pDMC1097-20.222kb. Black blocks show Tn*3* or Tn*3* sequences and the red triangles represent the Tn*3* inverted repeats (GGGGTCTGACGCTCAGTGGAACGAAAACTCACGTTAAG). Gray shadows show homologous regions. The large region of homology has 4335/4400 identical nucleotides, most differences are located within *tnpR*. The small region shows 320/320 identical nucleotides. The gray shadows are shown only in one of the inverted repeated elements in pDMC1097-20.222kb. The green blocks represent the IS*26* and the white triangles show the IS*26* inverted repeats (GGCACTGTTGCAAA). The DNA segment between the mobile elements (red) includes three pseudo genes, two of them corresponding to a serine hydrolase and the other a hypothetical protein. **(C)** Nucleotide sequence of the *res* regions of the pDMC1097-20.222kb mobile elements diagrammed in **(B)**. The numbers indicate the coordinates as shown in GenBank. The letters shown in color are the nucleotides that differ in Tn*1*, Tn*2*, and Tn*3* as described before (Partridge and Hall, 2005). Black, identical in Tn*1*, Tn*2*, and Tn*3.* Blue, identical to those in Tn*3*. Red, identical to Tn*2*. **(D)** Comparison of the genetic maps of pJHCMW1 and pNJST258N5. The black bars represent Tn*1331* and the orange and pink bars represent the DNA regions that include the inheritance functions. The coordinates of pJHCMW1 and pNJST258N5 have been modified from those in GenBank (accession numbers AF479774 and CP006924, respectively) to facilitate the comparison. Straight and crossed gray shadows represent direct and inverse orientation. The nucleotide sequences of the transposon share 99% identity. The numbers in the replication (REP) and Xer-recombination site (*mwr*) indicate the percent homology. **(E)** Comparison of the genetic maps of Tn*1331* and Tn*1331*-like structures. Black blocks represent Tn*1331* or Tn*1331* sequences and the red triangles represent the Tn*1331* inverted repeats, which are identical to those of Tn*3*. The IS*26* sequence present in Tn*1331*Δ:IS*26* is shown as a green block. The white triangles show the inverted repeats. The accession numbers of the sequences used for this figure are AF479774 (Tn*1331*), JF828150 (Tn*1331*Δ:IS*26*), and CP020840 (pBK13043-3).

The most studied *K. pneumoniae* plasmids are those that code for the complete conjugation machine, virulence factors, and mobile elements (de Toro et al., 2014). They are usually larger than 30-kb and can reach several hundred kb. Smaller *K. pneumoniae* plasmids, ∼25-kb or less, have attracted much less attention and the majority of the studies are limited to the analysis of their nucleotide sequences and a few complementary experiments. A notable exception is pJHCMW1, isolated from *K. pneumoniae* JHCK1, which caused several fatalities in a neonatal ward as a consequence of resistance to the antibiotic treatment (Tolmasky et al., 1986; Woloj et al., 1986; Xie et al., 2013). The pJHCMW1 plasmid includes the transposon Tn*1331* (nucleotides 3362-11654) and a 3361-nucleotides region that contains the genetic traits specific to inheritance and conjugation (Figure 1A) (Dery et al., 1997; Sarno et al., 2002). A BLAST analysis of this region showed homology to several plasmids. However, the homology was mostly confined to the ColE1-type replication region (Figure 2A) (Pasquali et al., 2005; Tolmasky et al., 2010; Ye et al., 2010; Fricke et al., 2011; Sun et al., 2013; Brantl, 2014; Porres-Osante et al., 2014; Calva et al., 2015; Cao et al., 2015; Lilly and Camps, 2015; Liu et al., 2015). The pJHCMW1 plasmid includes a functional *oriT* but lacks the genes coding for the relaxosome and the transferosome (Dery et al., 1997) (Figure 1A). Since *K. pneumoniae* strains usually harbor numerous plasmids, pJHCMW1 may occasionally be transferred to other strains when one or more co-resident plasmids contribute the appropriate helper functions. This was confirmed in a mobilization assay using a recombinant clone, pROXT1, generated by inserting the pJHCMW1 *oriT* region into pUC4K, and pRK2073 (Leong et al., 1982), a plasmid that includes the whole RK2 conjugation machinery. In a triparental mating including a recipient *E. coli* and *E. coli* strains carrying pROXT1 or pRK2073, the transfer frequencies of both plasmids were of the same order (1 and 3.3 × 10^–1^, respectively) (Dery et al., 1997). This experiment showed that if the appropriate machinery is coded for by another resident plasmid, pJHCMW1 can be mobilized in trans at frequencies comparable to those of the self-transmissible helper plasmid. Until recently, it was considered unusual that mobilizable plasmids include an *oriT* but not the genes coding for the specific relaxosome components. However, recent work on conjugation and mobilization of staphylococcal plasmids showed that many, some of them smaller than 5-kb, are transferred by relaxase-in trans mobilization, as it is the case for pJHCMW1 (O’Brien et al., 2015). Further studies showed that a large percentage of *S. aureus* plasmids include *oriT* mimics indicating that the number of plasmids with mobilization capabilities may be much higher than initially thought (Ramsay et al., 2016). The pJHCMW1 plasmid also possesses a Xer site-specific recombination site, *mwr*, which was thoroughly characterized (Figure 1A) (Tolmasky et al., 2000; Pham et al., 2002; Bui et al., 2006; Trigueros et al., 2009; Tran et al., 2010; Ramirez et al., 2014a). Xer site-specific recombination sites play roles in different processes like plasmid stability by promoting dimer resolution or plasmid evolution by formation and resolution of cointegrates and promoting integration of resistance genes and mobile elements (Zakharova et al., 2002; D’Andrea et al., 2009; Merino et al., 2010; Tran et al., 2012; Das et al., 2013; Midonet and Barre, 2014, 2016; Boyd et al., 2017; Cameranesi et al., 2018). They also participate in processes not involving plasmids such as chromosome dimer resolution or integration of IMEXs (integrative mobile elements exploiting Xer) (Barre et al., 2001; Sherratt et al., 2001; Midonet and Barre, 2014, 2016; Midonet et al., 2019).

While the partition systems required to ensure proper segregation of low copy number plasmids have been thoroughly studied in numerous systems (Baxter and Funnell, 2015), small high copy number plasmids received scarce attention because it was thought that random diffusion was sufficient to avoid generating plasmidless cells after cytokinesis. Few reports are available, and they all have in common that most plasmid molecules are located in the nucleoid-free regions. Localization of pJHCMW1, which has a calculated copy number of 24 when cells are about to divide, using fluorescence microscopy showed that while the bulk of the fluorescence was localized at the poles in nucleoid-free spaces, the shape and position of the fluorescent spots changed over time suggesting that the molecules were only partially restricted to the cell poles (Reyes-Lamothe et al., 2014). Furthermore, when the nucleoid-free spaces were increased using a mutant or treating wild-type cells with cephalexin, the fluorescence occupied all the space suggesting that most molecules are excluded from the chromosomal DNA mass in the nucleoid, pushing them to regions of low chromosomal density (Figure 1B) (Reyes-Lamothe et al., 2014). This process is similar to the exclusion suffered by ribosomes (Robinow and Kellenberger, 1994), whose diameter of gyration – a measure of the size of a polymer, numerically defined as the average of the squared distance of a point in a polymer from its center of mass – is significantly smaller (18 nm) than that of pJHCMW1 (265 nm) (Willumeit et al., 1997; Reyes-Lamothe et al., 2014). It was concluded that pJHCMW1 segregates by random diffusion but with most molecules occupying the nucleoid-free polar regions of the cell. Other studies found high copy number plasmids clustered at the cell poles or combining some molecules randomly distributed throughout the cell and some others clustered (Pogliano et al., 2001; Yao et al., 2007; Wang et al., 2016; Standley et al., 2019). Also, the possibility of active partition has been considered (Million-Weaver and Camps, 2014). However, it should be noted that some of these latter studies were carried out with plasmids with non-natural replicons such as pUC19. This is a derivative of the ColE1-type pMB1 replicon but in which the *rom* gene has been deleted, and a nucleotide substitution within RNA II alters its spatial structure in a temperature-dependent manner affecting the interaction between RNA II and the negative regulator RNA I (Minton et al., 1988; Lin-Chao et al., 1992). As a consequence, plasmids with this origin of replication have a copy number abnormally higher. This fact could be responsible for anomalous behavior of the plasmids. Nevertheless, all observations are consistent with plasmid localization by random diffusion and nucleoid exclusion. Future studies on other small plasmids will contribute to the understanding of their partition dynamics.

## Identification of Completely Sequenced Small *K. pneumoniae* Plasmids

A search for *K. pneumoniae* in the NCBI genome database^1^ following the link “Plasmid annotation report^2^ ” produced 172 plasmid sequences 25-kb or smaller. After further analysis of these sequences, those that had the same number of nucleotides and showed higher than 99% identity were considered to be the same plasmid (Table 1). The replication regions of pJHCMW1, pKPN2, pKlebB-k17/80, pIP843, pH205, pColEST258, and p15S had been characterized and shown to belong to the ColE1-type (Ramirez et al., 2014a). Alignment of the nucleotide sequences of these regions showed high conservation with three sectors with variability (nucleotides 182-208, 402-439, 565-609, Supplementary Figure S1A). Maximum divergence was estimated at 0.446 substitutions per site (Supplementary Figure S1B). Then, the replication regions of all plasmids were compared to the nucleotide sequences of the replication regions of each one of the seven plasmids named above. Among 172 plasmids, those that showed 50% or higher identity to at least one of the seven plasmids, were considered to possess a ColE1-type replication region (Table 1). Besides, some plasmids did not show 50% or more identity to the replication regions of either of the seven plasmids used as reference, but they are identified as ColE1-type in GenBank. These plasmids are also considered ColE1-type in Table 1. Other plasmids include putative replication proteins in the GenBank annotations, which suggest initiation of replication mechanism other than ColE1-type (Table 1). In a few other cases there are no indications of the replication mechanism, and in one case both a ColE1-type element and a potential replication protein have been identified (Table 1). The information shown in Table 1 indicates that there is a prevalence of the ColE1-type initiation of replication mechanism among sequenced small *K. pneumoniae* plasmids. To get insights into the evolutionary history of the small plasmids, an alignment-free sequence comparison among its complete sequence were done using Kmacs (Leimeister and Morgenstern, 2014). Subsequently, a hierarchical cluster analysis of studied plasmids was done based on the estimated symmetric distance (Supplementary Figure S2). The results suggest that the seven reference plasmids, pJHCMW1, pKPN2, pKlebB-k17/80, pIP843, pH205, pColEST258, and p15S, represent different lineages of the clustering or “subtypes.” The distributions on the clustering of key mobile elements, and resistance genes indicate that they seem to be shared mainly by two of the three “basal” clusters (Table 1), with only a few exceptions. These similarities may reflect vertical inheritance of the shared genes, however, since clustering method is not independent of gene composition we do not continue discussing this issue in order to avoid tautological thinking.

**TABLE 1:** Main characteristics of completely sequenced *K. pneumoniae* plasmids 25-kb or smaller.

**Plasmid name**	**Size (Kb)**	**Mobile elements and resistance genes^2^**	**Replication^3^**	**Accession number^1^/References**
pKPHS6	1.308		Rep protein	CP003228
p38547-1.476kb	1.476		ColE1-type	CP010388
pMRSN480738_1.6	1.551		ColE1-type	CP024465
Unnamed	1.556		Rep protein	CP023940
unnamed5	1.916		Rep protein	CP024520
pUMNturkey9_1	1.933		ColE1-type	CM003132
pKp_Goe_917-8	1.933		Rep protein	CP018439
pMYS	2.014		ColE1-type	CP006660 (Hudson et al., 2014)
unnamed4	2.155		ColE1-type	CP023906
pIGRK	2.348		Unknown	AY543071
pKP13a	2.459		ColE1-type	CP003996 (Ramos et al., 2014)
p38547-2.496kb	2.496		ColE1-type	CP010389
pIGMS31	2.52		Unknown	AY543072
unnamed6	2.723		ColE1-type	CP024495, CP024488
pMRSN480738_2.8	2.78		ColE1-type	CP024464
pCAV1042-2781	2.781		ColE1-type	CP018665
pCAV1596-2927	2.927		Unknown	CP011643
unnamed10	2.936		ColE1-type	CP024506
unnamed9	3.012		ColE1-type	CP024505
pB1020	3.174		ColE1-type	JQ319772
pKp04a, pKpN06-COL, pCAV1042-3223, pKP13b, unnamed4, unnamed5, unnamed8	3.223		ColE1-type	CP012991, CP014305, CP018666, CP024494, CP024487, CP024514, CP024519, CP024503, CP003994 (Ramos et al., 2014)
unnamed7	3.336		ColE1-type	CP024513
p187-3, pKPHS5	3.353		ColE1-type	CP025469, CP003227
unnamed6	3.377		ColE1-type	CP024512
pKPN7	3.478		ColE1-type	CP000652
unnamed4, unnamed5	3.514		ColE1-type	CP024493, CP024486, CP024511
pKp_Goe_641-5	3.541		ColE1-type	CP018739
pKp_Goe_917-7	3.559		ColE1-type	CP018446
p169	3.679	IS*Ecp1/bla*_CMY–__2_-like	ColE1-type	FM246880 (Verdet et al., 2009)
pB1021	3.692		ColE1-type	JQ319767
pCAV1344-3741, pCAV1193-3741	3.741		ColE1-type	CP011619, CP013321 (Sheppard et al., 2016)
pKPHS4	3.751		ColE1-type	CP003226
pKpn23412-4	3.777	IS*Kpn28*	ColE1-type	CP011316 (Becker et al., 2015)
unnamed4	3.808		ColE1-type	CP023915
unnamed5, unnamed6	3.825		ColE1-type	CP024197, CP024569, CP024527, CP024534, CP024541, CP024555, CP024562, CP024575
unnamed3	4		ColE1-type	CP023908
pKp_Goe_641-4	4.052		ColE1-type	CP018740
unnamed2, unnamed3	4.064		ColE1-type	CP024498, CP024518, CP024502
pKp_Goe_070-3	4.075		ColE1-type	CP018453
unnamed3	4.163		ColE1-type	CP023945
unnamed4	4.166		ColE1-type	CP023930
pKPN2	4.196		ColE1-type	AF300473 Den’mukhametov et al. (1997)
pKpn114	4.211		ColE1-type	EU932690
unnamed3	4.228	IS*1*-like	ColE1-type	CP024485
pKp_Goe_579-5	4.249		ColE1-type	CP018317
pKPN6	4.259		ColE1-type	CP000651
pCGH25	4.27	*qnrD1*	ColE1-type	JQ776509 (Zhang et al., 2013)
pKp_Goe_917-6, unnamed5	4.51		ColE1-type	CP018440, CP024196, CP024568, CP024526, CP024533, CP024540, CP024554, CP024561
unnamed4	4.66		ColE1-type/Rep protein	CP024195, CP024567, CP024525, CP024532, CP024539, CP024553, CP024560
_Plasmid_D_Kpneumoniae _MS6671	4.715		ColE1-type	LN824137
unnamed5	4.744		ColE1-type	CP014300
pKpn2312-5	4.831		ColE1-type	CP011315 (Becker et al., 2015)
p9701	4.84	IS*Ecp1*:IS*Kpn26-bla*_CMY–__2_-like	ColE1-type	FM246881 (Verdet et al., 2009)
pKP13c	5.065	IS*1R*^#^	Rep protein	CP003995 (Ramos et al., 2014)
pB1019	5.225		ColE1-type	JQ319775
pKp_Goe_917-5	5.234		ColE1-type	CP018444
pUUH239.1	5.247		ColE1-type	CP002473 (Sandegren et al., 2012)
pKlebB-k17/80	5.258		ColE1-type	AF156893 (Riley et al., 2001)
pKp_Goe_641-3	5.259		ColE1-type	CP018738
pCAV1042-5566	5.566		ColE1-type	CP018667
unnamed5, p69-4, p44-4	5.596		ColE1-type	CP023936, CP025460, CP025465
unnamed3	5.783		Rep protein	CP024492
unnamed2	6.139	IS*Ecp1*#/*bla*_OXA–__232_/*ereA3*#	Rep protein	CP016920
KP-plasmid2, pUCLAOXA232-1, plasmid4	6.141	IS*Ecp1*#/*bla*_OXA–__232_/*ereA3*#	ColE1-type	CP012755, CP012562, CP006802 (Doi et al., 2014; Kwon et al., 2016)
pOXA-232	6.328	IS*Ecp1*#/*bla*_OXA–__232_/*ereA3*#/IS*Ecp1*#	ColE1-type	CM009032 (Mancini et al., 2018)
unnamed2	6.657		ColE1-type	CP024491
pIP843	7.086	IS*Ecp1*/*bla*_CTX–M–__17_/IS*903-C*	ColE1-type	AY033516 (Cao et al., 2002)
pKP3-A	7.605	IS*Ecp1* plus 1305 bp/*bla*_OXA–__181_/*ereA3*#	ColE1-type	JN205800 (Potron et al., 2011)
Unnamed	8.187		ColE1-type	FJ042668 (Bojer et al., 2010)
pH205	8.197	IS*Ecp1*/*bla*_CMY–__36_	ColE1-type	EU331426 (Zioga et al., 2009)
tig00003569alt	8.364	Tn*5403*	ColE1-type	CP021700
p18-43_04	9.293		ColE1-type	CP023557 Osei Sekyere and Amoako (2017)
pIGMS32, pKp_Goe_917-4, pCAV1417-9294, pCAV1217-9294, pEA1509_B	9.294		ColE1-type	DQ298019, CP018445, CP018348, CP018672, CP009772 (Conlan et al., 2014)
unnamed4	9.326		ColE1-type	CP024574
CR14_p5	9.456	Tn*2*#/IS26/*ant(3^″^)-Ia*#/*aac(6′)-Ib*-T329/Tn*3*#	Unknown	CP015397
pKPN1482-5	9.51	Tn*6901*#	ColE1-type	CP020845 (Long et al., 2017)
pRYCKPC3.1	9.803	Tn*4401*#/IS*Kpn7*/ *bla*_KPC–__3_/IS*Kpn6*/Tn*4401*#	ColE1-type	GU386376
pMRSN480738_10.0	10.046		ColE1-type	CP024463
p69-3, p44-3	10.06		ColE1-type	CP025459, CP025464
unnamed3	10.061		ColE1-type	CP023944
plasmid 3	10.077		ColE1-type	CP017388
pKPN1481-5	10.373	Tn*2c*#/IS*26*/Tn*3*#/*aac(6′)-Ib*-T329/*ant(3^″^)-Ia*#	ColE1-type	CP020849 (Long et al., 2017)
pNJST258N5^5^	10.925	Tn*1/2/3*#/*bla*_TEM_/*bla*_OXA–__9_/*ant(3^″^)-Ia*/*aac(6′)-Ib*/Tn*1*#	ColE1-type	CP006924 (Deleo et al., 2014)
NY9_p6	11.09	IS*26*/Tn*3*#	ColE1-type	CP015391
pJHCMW1	11.354	Tn*3*#/*aac(6′)-Ib*-T329/*ant(3^″^)-Ia*/*bla*_OXA–__9_/Tn*3*#/*bla*_TEM–__1__a_	ColE1-type	AF479774 (Sarno et al., 2002)
unnamed4	11.972	Se.ma.I1 (group II intron; also called Kl.pn.I5)	ColE1-type	CP023932
pBK13043-3	11.984	*ant(3^″^)-Ia*#/*aac(6′)-Ib*-T329/Tn3#	ColE1-type	CP020840 (Long et al., 2017)
p2	12.207	Se.ma.I1 (group II intron; also called Kl.pn.I5)	ColE1-type	CP006658
unnamed3	12.273	IS*Ecp1*#/*bla*_OXA–__232_/ *ereA3*#/IS*Ecp1*#/*bla*_OXA–__232_/*ereA3*#	ColE1-type	CP023924
pKPN1481-4	12.376	IS*1R*	ColE1-type	CP020850 (Long et al., 2017)
p30684_3	12.399	IS*1294*#/IS*5075*#/IS*1294*#	ColE1-type	CP006921 (Deleo et al., 2014)
pKPN1482-4	12.54	Tn*6901* IR/—/Tn*6901* IRL	ColE1-type	CP020846 (Long et al., 2017)
pColEST258	13.636	Tn*2*#/IS*26*/*ant(3^″^)-Ia*#/*aac(6′)-Ib*-C329/Tn*3*#	ColE1-type	JN247853 Garcia-Fernandez et al. (2012)
p500_1420-13.838kb	13.838	Tn*2*#/IS*26*/*ant(3^″^)-Ia*#/*aac(6′)-Ib*-C329/Tn*3*#	ColE1-type	CP011984 (Wright et al., 2014)
p34618-13.841kb, pCAV1453-14, tig00000004, pBIC-1c, pKPN-294, pUHKPC07-13.841kb, pUHKPC33-13.841kb	13.841	Tn*2*#/IS*26*/*ant(3^″^)-Ia*#/*aac(6′)-Ib*-C329/Tn*3*#	ColE1-type	CP010394, CP018353, CP020112, CP022576, CP008832, CP009873, CP011988, CP011993 (Wright et al., 2014)
p4	13.848	IS*26*/*ant(3^″^)-Ia*#/*aac(6′)-Ib*-T329/Tn*3*#interTn*3*#/Tn*2*#/IS*26*#	Unknown	CM007853 (Ruppe et al., 2017)
p4	14.027	Tn*3*#/*aac(6′)-Ib*-T329/*ant(3^″^)-Ia*#/IS*26*/Tn*2*#	ColE1-type	CP019776 (Ruppe et al., 2017)
pNJST258N4	14.249	Tn*2c*#/*Tn1/2/3*#/*bla*_TEM–__1__a_	ColE1-type	CP006928 (Deleo et al., 2014)
ColE-LS6	14.709	IS*26*/Tn*2*#/Tn*3*#/*ant(3^″^)-Ia*#/*aac(6′)-Ib*-T329	ColE1-type	JX442973 (Villa et al., 2013)
pAAC154-a50	15.096	Tn*3*#/*aac(6′)-Ib*-T329/*ant(3^″^)-Ia*#/IS*26*/Tn*2*#	ColE1-type	CP008828, CP007728 (Snitkin et al., 2012)
CN1_p2, pAAC154-a9e	15.1	Tn*3*#/*aac(6′)-Ib*-T329/*ant(3^″^)-Ia*#/IS*26*/Tn*2*#	ColE1-type	CP015384, CP009877 (Conlan et al., 2014)
pAAC154	15.101	Tn*3*#/*aac(6′)-Ib*-T329/*ant(3^″^)-Ia*#/IS*26*/Tn*2*#	ColE1-type	JF828150 (Warburg et al., 2012)
pMNCRE69_1	15.27	IS*26*/Tn*2*#/Tn*3*#/*ant(3^″^)-Ia*#/*aac(6′)-Ib*-T329	ColE1-type	CP018425
tig00000004, tig00000601_pilon, pKPN-c8b	15.271	Tn*3*#/*aac(6′)-Ib*-T329/*ant(3^″^)-Ia*#/IS*26*/Tn*2*#	ColE1-type	CP021543, CP021717, CP021837, CP009778 (Conlan et al., 2014)
pMNCRE53_1	15.273	IS*26*/Tn*2*#/Tn*3*#/*aac(6′)-Ib*-T329/*ant(3^″^)-Ia*#	ColE1-type	CP018433
pKp_Goe_795-4	16.971		Unknown	CP018459
KPN207_p4	18.623	*aac(6′)-Ib*Other#/*ant(3^″^)-Ia*#/IS*26*/Tn*2*#/Tn*3*#/*aac(6′)-Ib*-T329/*ant(3^″^)-Ia*#/IS*26*/Tn*2*#	ColE1-type	LT216440
pMNCRE78_1	18.89	IS*26*/Tn*2*#/Tn*3*#/*aac(6′)-Ib*-T329/*ant(3^″^)-Ia*#/IS*26*/Tn*2*#	ColE1-type	CP018431
pDMC1097-20.222kb^4^	20.222	Tn*1/2/3*-like#/IS*26*/ Tn*2*#/Tn*2*#/IS*26*/Tn*1*,*2*,*3*-like#	ColE1-type	CP011979 (Wright et al., 2014)
pKPN1481-3	20.38	IS*Kpn28*	ColE1-type	CP020852 (Long et al., 2017)
pSLMT	21.138	IS*Kpn31*#/Tn*4401*#/IS*Kpn7*/ *bla*_KPC–__2_/IS*Kpn6*/Tn*4401*#/ IS*Kpn31*#/Tn*5403*#	ColE1-type	HQ589350
tig00000005	22.062	IS*26*#/Tn*1/2/3*#/ *bla*_TEM–__1__a_/Tn*4401*#/ IS*Kpn7*/*bla*_KPC–__3_/IS*Kpn6*/Tn*4401*#	ColE1-type	CP020075
unitig_5	22.632	IS*Kpn6*#/Tn*4401*#/Tn*2*#/IS*26*/ *ant(3^″^)-Ia*#/*aac(6′)-Ib*-T329/Tn*1*#/Tn*4401*#/IS*Kpn7*/ *bla*_KPC–__3_/IS*Kpn6*#	ColE1-type	CP021756
tig00000005	22.633	Tn*1*#/Tn*4401*#/IS *Kpn7*/*bla*_KPC–__3_/IS*Kpn6* /Tn*4401*#/Tn*2*#/IS*26*/*ant (3^″^)-Ia*#/*aac(6′)-Ib*-T329	ColE1-type	CP021548
p15S	23.753	Tn*2*#/IS*26*/*ant(3^″^)-Ia*#/ *aac(6′)-Ib*-T329/Tn*3*#/ Tn*4401*#/IS*Kpn7*/*bla*_KPC–__2_/ IS*Kpn6*/Tn*4401*#/	ColE1-type	FJ223606 (Gootz et al., 2009)
tig00000007_pilon	24.749	Tn*4401*#/IS*Kpn6*/*bla*_KPC–__3_/ IS*Kpn7*/Tn*4401*#/Tn*2*#/IS*26*/ *ant(3^″^)-Ia*#/*aac(6′)-Ib*-T329/Tn*3*#	ColE1-type	CP021860

*^1^Plasmids were considered identical and listed together if they have the same number of nucleotides and identity >99%. ^2^The nucleotide sequences were analyzed using the Multiple Antibiotic Resistance Annotator (MARA) and database (Partridge and Tsafnat, 2018). # indicates that an incomplete copy of the feature is present. Annotations of partial features may not distinguish correctly between particular variants. ^3^The initiation of replication mechanisms were identified by the annotations in GenBank, or homology to the origin of replication regions of pJHCMW1, pKPN2, pKlebB-k17/80, pIP843, pH205, pColEST258, and p15S. Rep protein indicates that an ORF with the characteristics of a replication protein was annotated in GenBank. In one case the annotations included features of ColE1 replication and a replication protein. ^4^In each of the two inverted repeated copies of the Tn1/2/3# copies in the plasmid pDMC1097-20.222kb there is a fragment of *bla*_*TEM–*__1_ (see Figure 2B). ^5^The nucleotide 329 in the sequence CP006924 in GenBank is shown as “N.”*

## Antibiotic Resistance Genes and Their Inclusion Within Mobile Elements

The presence of antibiotic resistance genes and mobile elements were searched using the Multiple Antibiotic Resistance Annotator (MARA) and database (Partridge and Tsafnat, 2018) (Table 1). Seven plasmids include genes coding for KPC enzymes while three and one carry genes coding for CMY and CTX-M enzymes, respectively (Table 1). The *bla*_CMY_ genes are located adjacent to insertion sequences, IS*Ecp1* in two cases and ISEcp1:IS*Kpn26* in another (Table 1) (Verdet et al., 2009). The *bla*_CTX–M__15_ gene, present in pIP843, is located between the insertion sequences IS*Ecp1* and IS*903-C*, and the *bla*_KPC_ genes are associated to Tn*4401* or related structures (Table 1) (Cao et al., 2002; Gootz et al., 2009). Five plasmids include *bla*_OXA–__48_-like genes. Four of them include *bla*_OXA–__232_ and one *bla*_OXA–__181_. These genes code for proteins that differ at a single amino acid at position 214, R214 in OXA-181 and S214 in OXA-232 (Potron et al., 2013). These genes are found flanked by IS*Ecp1* and an incomplete copy of the erythromycin resistance *ereA* gene, followed by an imperfect second copy of the IS*Ecp1* right inverted repeat (Table 1) (Potron et al., 2011).

Four plasmids harbor the complete *bla*_TEM–__1_ gene, in two of them within a Tn*3*-like structure and in pNJST258N5 and pJHCMW1 within Tn*1331*, a transposon described in detail below. The pDMC1097-20.222kb harbors a truncated *bla*_TEM–__1_ gene (Figure 2B). This plasmid includes inverted repeated copies of a transposon-like structure that could have been generated after insertion of IS*26* within a Tn*3-*like transposon followed by a recombination process mediated by the insertion sequence (Figure 2B). Analysis of the *res* region of the mobile elements present in pDMC1097-20.222kb, which includes nucleotides that differ in Tn*1*, Tn*2*, and Tn*3* (Partridge and Hall, 2005) showed that in one portion the key nucleotides correspond to those in Tn*3* and in another one the key nucleotides are those found in Tn*2* (see Figure 2C).

The *aac(6′)-Ib* gene, which can be found in two main variants that differ in one amino acid, L or S (T-329 or C-329, respectively, in Table 1) (Rather et al., 1992; Ramirez and Tolmasky, 2010), was found in two plasmids within a complete copy of Tn*1331* (pJHCMW1) and a transposon with 99% identity with Tn*1331* (pNJST258N5) and in 21 plasmids within Tn*1331* derivatives associated to IS*26* (see below). The *aac(6′)-Ib* gene codes for an acetyltransferase that mediates resistance to numerous aminoglycosides including amikacin (variant T-329) or gentamicin (variant C-329) (Ramirez et al., 2013). This gene, originally identified as part of the transposon Tn*1331* within pJHCMW1 (Figure 1) and the *Serratia marcescens* pAZ007 (Tolmasky et al., 1986; Woloj et al., 1986; Tolmasky and Crosa, 1987; Tran van Nhieu and Collatz, 1987; Nobuta et al., 1988; Partridge, 2015), is the most prevalent in amikacin-resistant Gram-negative clinical isolates and as such it has been the subject of numerous studies to characterize the AAC(6′)-Ib enzyme and to inhibit its effects (Shmara et al., 2001; Pourreza et al., 2005; Lombes et al., 2008; Maurice et al., 2008; Vetting et al., 2008; Soler Bistue et al., 2009; Lin et al., 2014; Li et al., 2015; Lopez et al., 2015; Chiem et al., 2016, 2018; Tran et al., 2018). It may be worthwhile to mention the finding in a *K. pneumoniae* isolate of a variant of Tn*1331*, Tn*6238*, that includes the *aac(6′)-Ib-cr* gene, which has two nucleotide changes and codes for an enzyme form that catalyzes acetylation of ciprofloxacin (Quiroga et al., 2015). The only other completely sequenced *K. pneumoniae* plasmid 25-kb or smaller that includes a close relative to Tn*1331* (99% identity, called Tn*1331* in this review) in its structure is pNJST258N5, recently found in a carbapenem-resistant sequence type (ST) 258 isolate from a patient with urinary tract infection (Chen et al., 2014b; Deleo et al., 2014). A comparison of the pJHCMW1 and pNJST258N5 sequences shows that although they share the Tn*1331* transposon, their backbones share homology only at the replication regions and Xer site-specific recombination sites (Figure 2D). Furthermore, these DNA regions are found in opposite orientations with respect to Tn*1331*. These structural properties suggest that both plasmids were generated independently by insertion of Tn*1331* into two different plasmids. The transposon Tn*1331* is a derivative of Tn*3* with the addition of a segment including three resistance genes, *aac(6′)-Ib* (also known as *aacA4*), *ant(3^″^)-Ia* (also known as aadA1) (Ramirez and Tolmasky, 2010), and *bla*_OXA–__9_. This region is flanked by 520-bp direct repeats and its structure resembles the variable region of integrons (Tolmasky, 1990; Tolmasky and Crosa, 1993; Ramirez et al., 2008). A detailed description of this segment has been included in previous articles (Tolmasky, 2000; Sarno et al., 2002; Ramirez et al., 2008; Ramirez and Tolmasky, 2010). A derivative of Tn*1331*, named Tn*1331*Δ:IS*26*, that has an insertion of IS*26* and a deletion that includes part of *ant(3^″^)-Ia*, the complete *bla*_OXA–__9_, and part of *bla*_TEM–__1_ (Figure 2E) was first described in pAAC154, and it was later found in several plasmids (Table 1) (Warburg et al., 2012; He et al., 2016). Interestingly, in most cases, the usual flanking direct repeats at the sites of insertion of Tn*1331*Δ:IS*26* are missing, probably due to IS*26*-mediated processes (Iida et al., 1984; Dawes et al., 2010; Harmer and Hall, 2015; He et al., 2015). A truncated version of Tn*1331*Δ:IS*26* that lacks the region encompassing IS*26* and the terminal fraction of Tn*1331* was found in the 11,984-nucleotide pBK13043-3 (Table 1 and Figure 2E). The recently described precise excision process mediated by IS*26* is probably responsible for the generation of the structure found in this plasmid (Harmer and Hall, 2015). The *aac(6′)-Ib* gene was also found associated with other Tn*3* and IS*26* structures (see Table 1). Although other derivatives of Tn*1331* were identified (Tolmasky et al., 1988; Poirel et al., 2006; Rice et al., 2008), none of these elements were detected in fully sequenced *K. pneumoniae* plasmids 25-kb or smaller.

## Concluding Remarks

Plasmids are known to host genes coding for virulence factors and antibiotic resistance. These genes are usually located within elements that disseminate at the molecular level. As a consequence, the interplay between dissemination between DNA molecules and the ability of plasmids to disseminate at the cellular level practically erase all barriers for genes to reach virtually all bacteria. Recent research about the genetic characteristics of *K. pneumoniae* pathogenic strains shows that this bacterium usually harbors numerous plasmids that can shape its properties as well as the nature of the diseases caused. However, within the variety of plasmids found in this bacterium, most of the attention has been directed to large plasmids that can accumulate numerous genetic determinants, mobile elements, and the information of self-transmission. A revision of the information available shows that smaller plasmids, which tend to possess higher copy numbers, can also be active participants in shaping *K. pneumoniae* strains and their evolution. They can disseminate at the cellular level by mobilization, contribute to plasmid evolution, and enhance gene expression by gene dosage. Despite the small size and limited number of genes they harbor, important resistance genes are usually present in most instances as part of transposons and integron-like structures. All these properties make them important contributors to shaping the biological properties of *K. pneumoniae* and other bacteria of interest to human health including multidrug resistance.

## Data Availability Statement

Publicly available datasets were analyzed in this study. This data can be found here: http://www.ncbi. nlm.nih.gov/genome/plasmids.

## Author Contributions

All the authors contributed equally to the writing of this review.

## Conflict of Interest

The authors declare that the research was conducted in the absence of any commercial or financial relationships that could be construed as a potential conflict of interest.

## Footnotes

1 https://www.ncbi.nlm.nih.gov/genome/

2 https://www.ncbi.nlm.nih.gov/genome/plasmids/815?
