# Supplementary material for: Small Klebsiella pneumoniae Plasmids: Neglected Contributors to Antibiotic Resistance
Source: Front Microbiol. 2019 Sep 20;10:2182. doi: 10.3389/fmicb.2019.02182 (PMC6764390; doi:10.3389/fmicb.2019.02182)
Supplement: Supplementary file 1 [file Presentation_1.PPTX]

## Slide 1
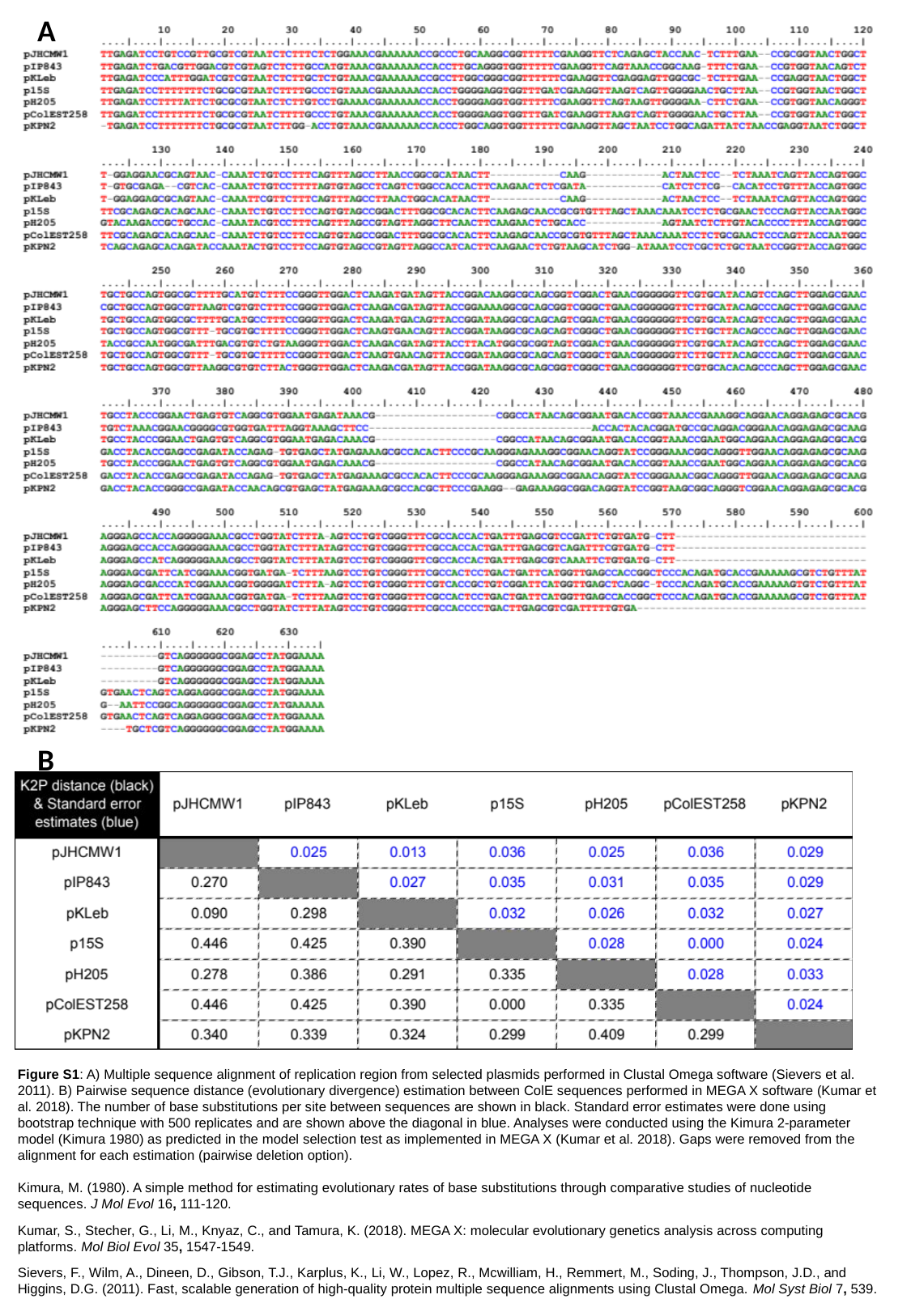

A
B
Figure S1: A) Multiple sequence alignment of replication region from selected plasmids performed in Clustal Omega software (Sievers et al. 2011). B) Pairwise sequence distance (evolutionary divergence) estimation between ColE sequences performed in MEGA X software (Kumar et al. 2018). The number of base substitutions per site between sequences are shown in black. Standard error estimates were done using bootstrap technique with 500 replicates and are shown above the diagonal in blue. Analyses were conducted using the Kimura 2-parameter model (Kimura 1980) as predicted in the model selection test as implemented in MEGA X (Kumar et al. 2018). Gaps were removed from the alignment for each estimation (pairwise deletion option).
Kimura, M. (1980). A simple method for estimating evolutionary rates of base substitutions through comparative studies of nucleotide sequences. J Mol Evol 16, 111-120.
Kumar, S., Stecher, G., Li, M., Knyaz, C., and Tamura, K. (2018). MEGA X: molecular evolutionary genetics analysis across computing platforms. Mol Biol Evol 35, 1547-1549.
Sievers, F., Wilm, A., Dineen, D., Gibson, T.J., Karplus, K., Li, W., Lopez, R., Mcwilliam, H., Remmert, M., Soding, J., Thompson, J.D., and Higgins, D.G. (2011). Fast, scalable generation of high-quality protein multiple sequence alignments using Clustal Omega. Mol Syst Biol 7, 539.
