# Supplementary material for: Small Klebsiella pneumoniae Plasmids: Neglected Contributors to Antibiotic Resistance
Source: Front Microbiol. 2019 Sep 20;10:2182. doi: 10.3389/fmicb.2019.02182 (PMC6764390; doi:10.3389/fmicb.2019.02182)
Supplement: Supplementary file 2 [file Presentation_2.PPTX]

## Slide 1
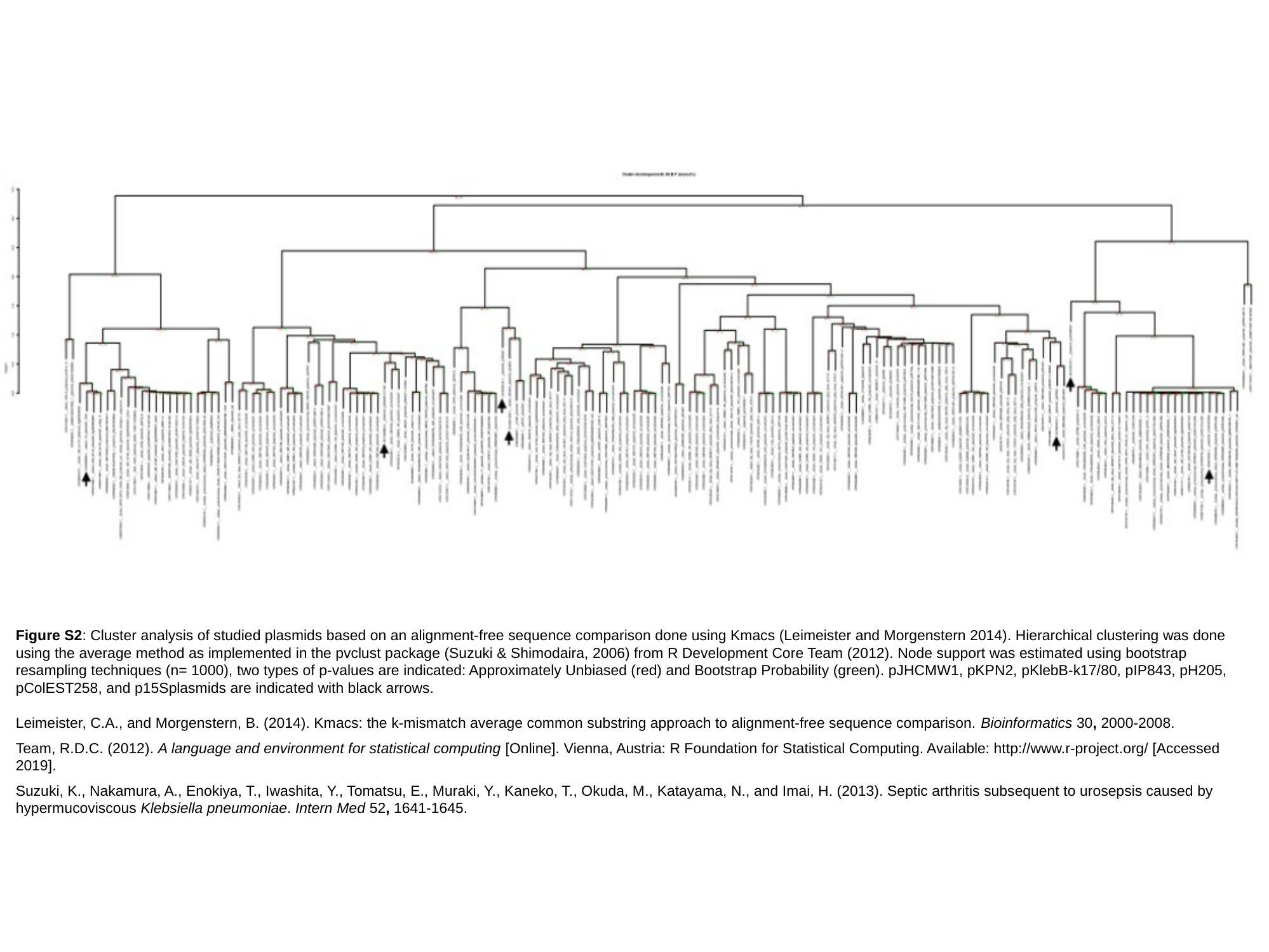

Figure S2: Cluster analysis of studied plasmids based on an alignment-free sequence comparison done using Kmacs (Leimeister and Morgenstern 2014). Hierarchical clustering was done using the average method as implemented in the pvclust package (Suzuki & Shimodaira, 2006) from R Development Core Team (2012). Node support was estimated using bootstrap resampling techniques (n= 1000), two types of p-values are indicated: Approximately Unbiased (red) and Bootstrap Probability (green). pJHCMW1, pKPN2, pKlebB-k17/80, pIP843, pH205, pColEST258, and p15Splasmids are indicated with black arrows.
Leimeister, C.A., and Morgenstern, B. (2014). Kmacs: the k-mismatch average common substring approach to alignment-free sequence comparison. Bioinformatics 30, 2000-2008.
Team, R.D.C. (2012). A language and environment for statistical computing [Online]. Vienna, Austria: R Foundation for Statistical Computing. Available: http://www.r-project.org/ [Accessed 2019].
Suzuki, K., Nakamura, A., Enokiya, T., Iwashita, Y., Tomatsu, E., Muraki, Y., Kaneko, T., Okuda, M., Katayama, N., and Imai, H. (2013). Septic arthritis subsequent to urosepsis caused by hypermucoviscous Klebsiella pneumoniae. Intern Med 52, 1641-1645.
